# Tuberculin Skin Test Distribution following a Change in BCG Vaccination Policy

**DOI:** 10.1371/journal.pone.0086419

**Published:** 2014-01-23

**Authors:** Sei Won Lee, Soo Yeon Oh, Jin Beom Lee, Chang Min Choi, Hee Jin Kim

**Affiliations:** 1 Department of Pulmonary and Critical Care Medicine, Asan Medical Center, University of Ulsan College of Medicine, Seoul, Republic of Korea; 2 The Korean Institute of Tuberculosis, Osong, Republic of Korea; Institut de Pharmacologie et de Biologie Structurale, France

## Abstract

**Background:**

Epidemiologic data regarding tuberculin skin test (TST) responses are an important basis for TB control strategies. This study analyzed TST responses in Korea, which experienced a rapid change in BCG vaccination status.

**Methods:**

TST responses in young adults were examined over 5 years. Participants with active TB lesions were excluded.

**Results:**

A total of 5,552 participants were enrolled with median age of 21 years. When an induration diameter ≥10 mm was used as the criterion for a positive test, TST positivity fell (from 28.0% in 2005 to 15.3% in 2009); however, they remained steady when the criterion was ≥15–20 mm. A positive TST was associated with a personal or family of TB, the presence of a Bacille Calmette-Guérin (BCG) scar, and age (odds ratio [95% confidence interval] = 4.03 [2.61–6.22], 2.91 [1.80–4.71], 1.50 [1.31–1.72], and 1.15 [1.09–1.20], respectively). Among these factors, the decrease of participants with BCG scars was the most prominent change, which appeared to be associated with the change of TST positivity rate.

**Conclusion:**

Overall, the rate of TST positivity in Korea decreased. However, this trend seems associated with the change of BCG vaccination strategy rather than successful control of LTBI. This study showed that change in BCG vaccination strategy can have great impact on TB epidemiologic survey based on TST.

## Introduction

The number of new tuberculosis (TB) cases has been falling since 2002; however, there were still 5.7 million new cases worldwide in 2010 [1]. The management of latent TB infection (LTBI), like that of active TB, become an essential part of any TB control strategy [2]. Recently, interferon-gamma release assays (IGRA) has recently been adopted to detect LTBI, which has several advantages [3], [4]. However, the tuberculin skin test (TST) is still the main method used to diagnose LTBI in mass screening, because IGRAs are costly and require laboratory facilities. Tuberculin responses are affected by factors such as vaccination with Bacille Calmette-Guérin (BCG) and exposure to non-tuberculous mycobacterium (NTM) infections; resulting in low specificity [5]. It is essential that we know the basal positive TST rate, its trend, and the effects of other factors if we are to accurately interpret TST results within the patient population.

The Republic of Korea is in a particularly delicate situation regarding TB control. The Korean government has made great efforts to control TB; however, Korea is still classed as having an “intermediate” burden, which means more elaborate strategies are needed. TB outbreaks are still reported occasionally [6], [7]. Mass BCG vaccinations are still offered in Korea, although many countries with a low TB burden have discontinued them. Infant are usually vaccinated with BCG at their birth. Before 1997, Koreans showing a negative TST at 12-years-of-age were revaccinated with BCG; however, in 1997 the WHO recommended that the revaccination program be abandoned [8]–[10]. This change in recommendation had been adapted to Korean vaccination strategy stepwise. Because of this, individuals may have been vaccinated with BCG from none to twice (as an infant and at 12 years-of-age), depending on their year of birth and the area in which they grew up. This change may have had a great impact on TST surveillance results, but it has not been evaluated well.

The objective of the present study was to: 1) obtain the basal rate and trend of TST positivity in Korea and ascertain whether LTBI is decreasing or not; and 2) examine which factors affect tuberculin responses.

## Methods

### Ethics Statement

The study protocol was approved by the Institutional Review Board of the Korean Institute of Tuberculosis and was consistent with the principles of the Declaration of Helsinki. Participants provided their written informed consents to participate in this study.

### Participants

We enrolled a term of entire intake of military conscripts per year in one Korean Reserve Force Battalion in 2005 and between 2008 and 2011. Military conscripts were recruited from across Korea. Each participant was given a chest X-ray (CXR) and blood tests (including a human immunodeficiency virus (HIV) test) when they began their training (the next day of their joining into army). Those with a CXR consistent with active TB or those that were HIV-positive were excluded. The initial study was planned to run for five consecutive years; however, the survey could not be undertaken in 2006 and 2007 due to a lack of cooperation from the Korean army. Participants were categorized into three groups according to their year of birth to analyze the association between the number of BCG scars and tuberculin responses. The national strategy of vaccinating 12-year-olds did not officially apply to those born after 1985 as the program was abandoned in 1997.

### Tuberculin Skin Test and Study Design

All participants provided informed consent. All received a one-step TST using 0.1 ml (2 TU) of purified protein derivative (RT23; Statens Serum Institute, Copenhagen, Denmark) according to standard procedures [11]. All TSTs were performed on the second day after entry into the army. The largest transverse diameter of the induration was measured 48−72 hr after the injection; a positive TST was defined as an induration with a diameter ≥5 mm, ≥10 mm, ≥15 mm, or ≥20 mm [2]. All tests were performed by four trained nurses. The investigator and the nurses were blinded to the patients’ demographic characteristics.

### Statistical Analysis

The relationship between clinical characteristics and TST results was evaluated using the χ2 test for categorical variables and by logistic regression analysis for continuous variables. When analyzing this relationship, a positive TST was defined as an induration with a diameter ≥10 mm due to the large number of subjects with BCG scars [12]. Backward selection was performed to exclude multi-colinearity from the multivariate analyses. All statistical analyses were conducted using PASW software (v18.0; SPSS, Inc., Chicago, IL, USA).

## Results

### Baseline Characteristics

A total of 5,552 participants were enrolled in the study (between 778 and 1,483 participants were enrolled per year). All were male (median age, 20 years; range, 18–29 years). In 2005, 12.6% of participants had two BCG scars; however, this percentage decreased markedly in 2008–2011 (0.4–2.5%). A third of participants (1,857; 33.4%) had no BCG scar. Eighty four (1.8%) participants reported a history of TB and 42 (1.3%) reported a family history of TB. About half of the participants were smokers (Table 1).

**Table 1:** Baseline characteristics of participants.

Characteristics	2005(n = 778)	2008(n = 1,092)	2009(n = 1,238)	2010(n = 1,483)	2011(n = 961)	Total(n = 5,552)
Age, median (range)	20 (19–26)	21 (18–29)	20 (18–28)	20 (18–29)	20 (18–27)	20 (18–29)
Male, n (%)	778 (100)	1,102 (100)	1,238 (100)	1,483 (100)	961 (100)	4,784 (100)
Number of BCG scars, n (%)						
2	98 (12.6)	27 (2.5)	13 (1.1)	6 (0.4)	15 (1.6)	159 (2.9)
1	533 (68.5)	658 (60.3)	703 (56.8)	975 (65.7)	667 (69.4)	3,536 (63.7)
0	147 (18.9)	407 (37.3)	522 (42.2)	502 (33.9)	279 (29.0)	1,857 (33.4)
Previous history of TB, n (%)	13 (1.1)	7 (0.6)	50 (4.0)	12 (0.8)	15 (1.6)	84 (1.8)
Family history of TB, n (%)	17 (2.2)	6 (0.5)	12 (1.0)	19 (1.3)	25 (2.6)	42 (1.3)
Smoker*, n (%)						
Current				723 (48.8)	619 (64.4)	1,342 (54.9)
Former				74 (5.0)	24 (2.5)	98 (4.0)
Never				686 (46.3)	318 (33.1)	1,004 (41.1)

* Data available for 2010–2011 only.

### TST Positivity Rates during 5 Years

**Figure 1:**
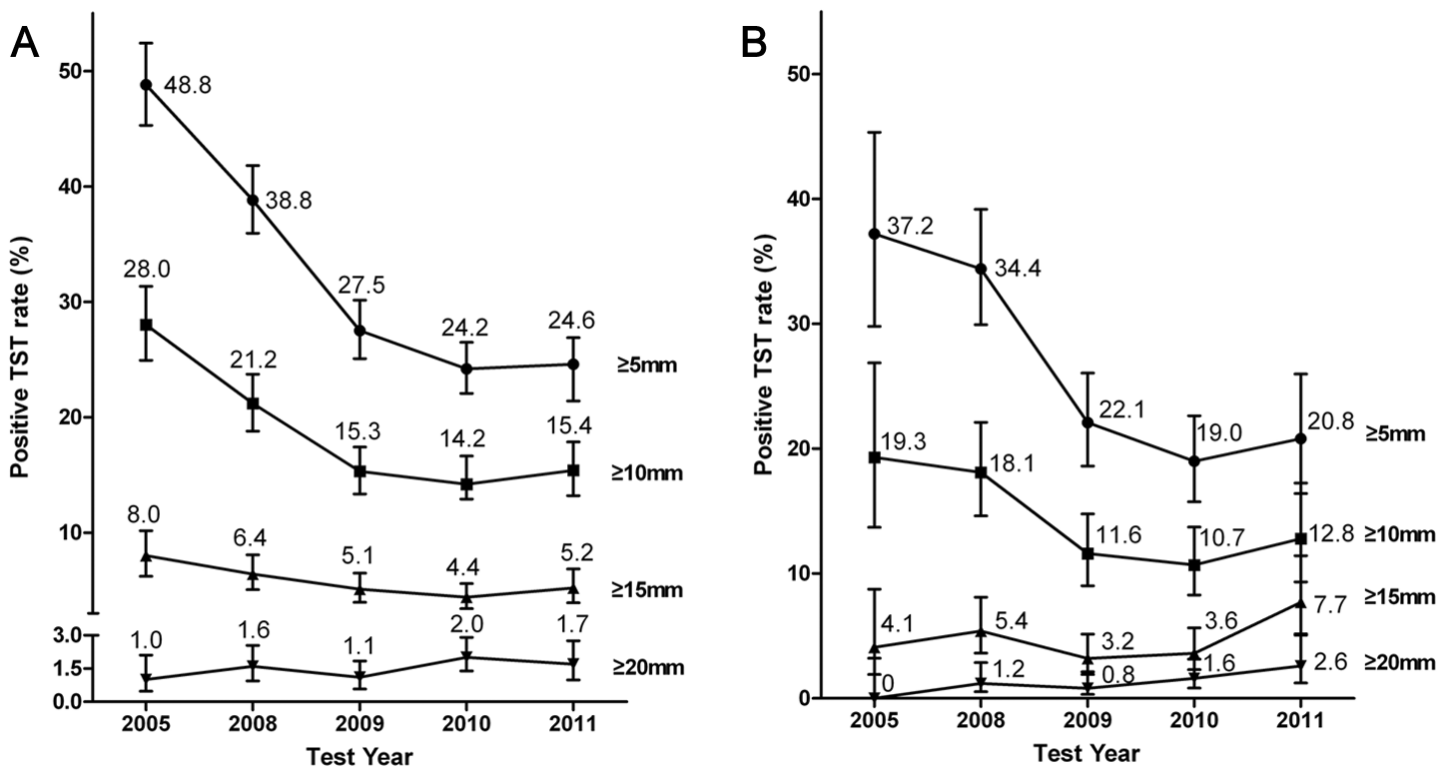
TST positivity over 5 years. (A) TST positivity rates for all participants. (B) TST positivity rates for participants with no BCG scar or history of TB. A positive TST was defined as induration with a diameter of ≥5 mm, ≥10 mm, ≥15 mm, or ≥20 mm. Error bar indicates 95% confidence interval. TST = Tuberculin skin test.

The TST positivity rates (according to the diameter of induration) for all participants in 2005 were as follows: 48.8% (≥5 mm), 28.0% (≥10 mm), 8.0% (≥15 mm) and 1.0% (≥20 mm). These rates decreased in 2008, but remained steady from 2009 to 2011. Meanwhile, the proportion of participants with induration ≥20 mm remained steady at 1.0–2.0% over study period (2005–2011). Similar trends were noted in participants without a BCG scar and a history of TB, who showed TST positivity rates by the definition of induration size ≥5 mm and ≥10 mm. However, TST positivity rates of indurations ≥15 mm and ≥20 mm in diameter were steady or showed a slight increase during the study period (3.2–7.7% and 0–2.6%, respectively (Figure 1)).

### Factors Associated with TST Results

Univariate analysis identified age, year of birth, the presence of a BCG scar, and a personal or family history of TB as being all associated with induration ≥5, 10 and 15 mm in diameter, except the association between BCG scar and induration ≥15 mm. An induration ≥20 mm in diameter was only associated with personal history of TB. Smoking was not associated with a TST response (Table 2). Multivariate analysis identified a personal or family history of TB, the presence of a BCG scar, and the year of birth as being associated with a positive TST (induration ≥10 mm in diameter) with odds ratios (ORs) of 3.91 (95% confidence interval [CI], 2.53−6.06), 2.96 (95% CI, 1.82−4.80), 1.37 (95% CI, 1.19−1.58) and 0.86 (95% CI, 0.84−0.89), respectively. Age was excluded from the final analysis due to multi-colinearity (Table 3).

**Table 2:** TST results according to different characteristics.

Characteristic	TST result, n (%)			
	≥5 mm	≥10 mm	≥15 mm	≥20 mm
Age at examination				
18–20 (n = 3,266)	836 (25.6)	461 (14.1)	140 (4.3)	40 (1.2)
21–23 (n = 2,055)	803 (39.1)	468 (22.8)	141 (6.9)	37 (1.8)
24–26 (n = 199)	84 (42.2)	58 (29.1)	23 (11.6)	6 (3.0)
27–29 (n = 32)	17 (53.1)	10 (31.3)	6 (18.8)	1 (3.1)
* P*-value	<0.001	<0.001	<0.001	0.08
Year of birth				
1980–84(n = 202)	122 (32.8)	81 (40.1)	28 (13.9)	4 (2.0)
1985–88(n = 1,914)	766 (40.0)	435 (22.7)	137 (7.2)	33 (1.7)
1989–93(n = 3,436)	852 (24.8)	481 (14.0)	145 (4.2)	47 (1.4)
* P*-value	<0.001	<0.001	<0.001	0.51
Number of BCG scars				
2 (n = 159)	102 (64.2)	48 (30.2)	11 (6.9)	0 (0)
1 (n = 3,536)	1159 (32.8)	684 (19.3)	208 (5.9)	56 (1.6)
0 (n = 1,857)	479 (25.8)	265 (14.3)	91 (4.9)	28 (1.5)
* P*-value	<0.001	<0.001	0.25	0.27
TB history				
Present (n = 97)	59 (60.8)	48 (49.5)	23 (23.7)	5 (5.2)
Absent (n = 5,455)	1681 (30.8)	949 (17.4)	287 (5.3)	79 (1.4)
* P*-value	<0.001	<0.001	<0.001	0.003
Family TB history				
Present (n = 79)	44 (55.7)	38 (48.1)	16 (20.3)	3 (3.8)
Absent (n = 5,473)	1,696 (31.0)	959 (17.5)	594 (5.4)	81 (1.5)
* P*-value	<0.001	<0.001	<0.001	0.09
Smoker				
Current (n = 1,342)	344 (25.6)	215 (16.0)	65 (4.8)	24 (1.8)
Former (n = 98)	20 (20.4)	11 (11.2)	3 (3.1)	3 (3.1)
Never (n = 1,004)	231 (23.0)	133 (13.2)	47 (4.7)	19 (1.9)
* P*-value	0.22	0.10	0.72	0.67

TST: tuberculin skin test.

**Table 3:** Factors associated with a positive TST (induration diameter ≥10 mm) by multivariate analysis.

	Odds ratio (95% CI)	*P*-value
TB history	3.91 (2.53−6.06)	<0.001
Family TB history	2.96 (1.82−4.80)	<0.001
Number of BCG scars*	1.37 (1.19−1.58)	<0.001
Year of birth*	0.86 (0.84−0.89)	<0.001

CI: confidence interval.

* Odds ratio as increase of one BCG scar or born one year later.

### TST Results According to Year of Birth and Age

**Figure 2:**
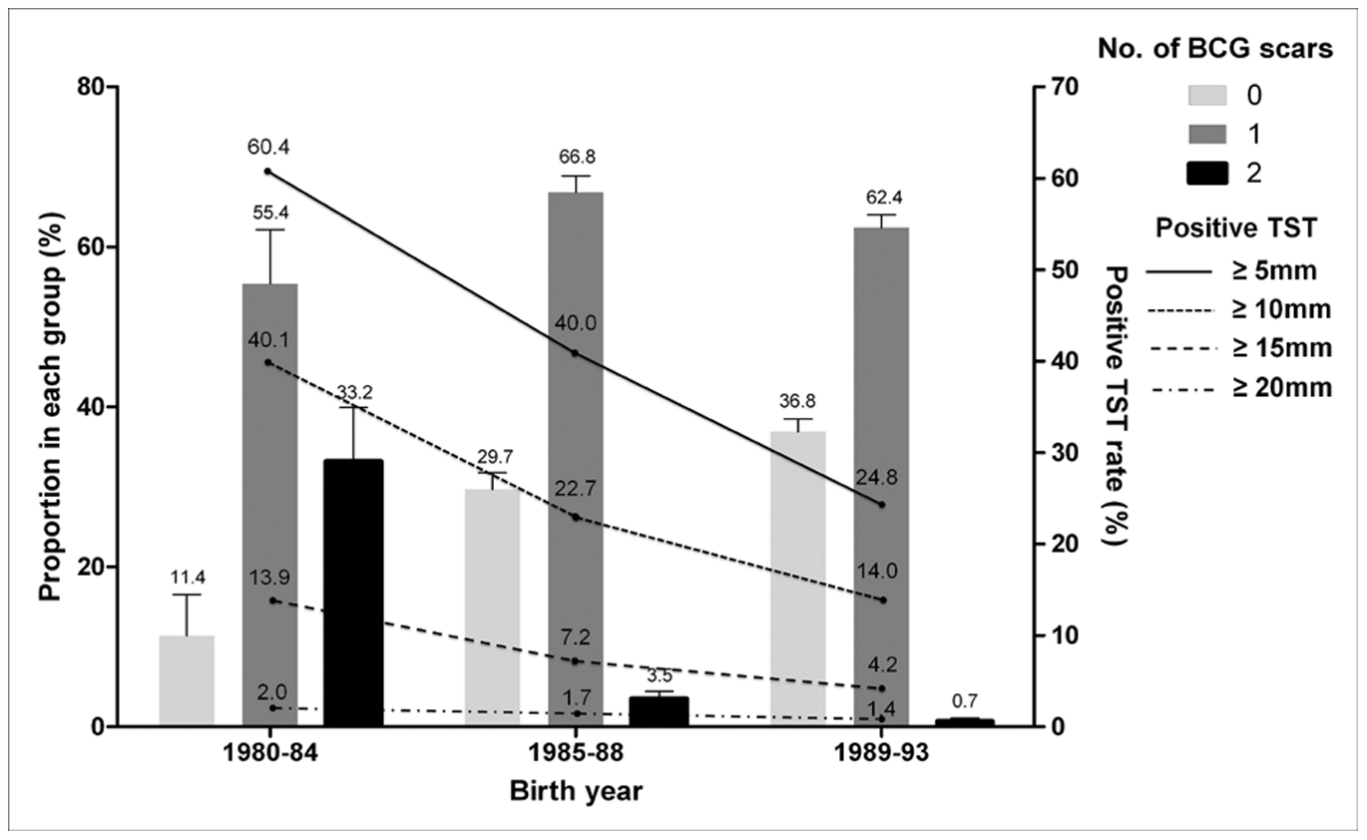
Number of BCG scars and TST-positive rates according to year of birth. BCG = Bacille Calmette-Guérin.

Participants were categorized into three groups according to year of birth (1980–84, n = 202; 1985–88, n = 1,914; and 1989–93, n = 3,436). The number of BCG scars decreased in participants born after 1985: 33.2% (67/202) of those born before 1984 had two BCG scars. However, the percentage of participants born after 1985 that had two scars fell to 0.7–3.5%. Accordingly, the proportion of participants without a BCG scar that were born between 1980 and 1984 was 11.4% (23/202), increasing to 36.8% (1266/3436) for those born between 1989 and 1993. Also, TST positivity rates decreased in those born after 1985. This trend was mirrored by the induration ≥5 mm, ≥10 mm and ≥15 mm, but the proportion of participants with induration ≥20 mm was almost stationary (Figure 2).

**Figure 3:**
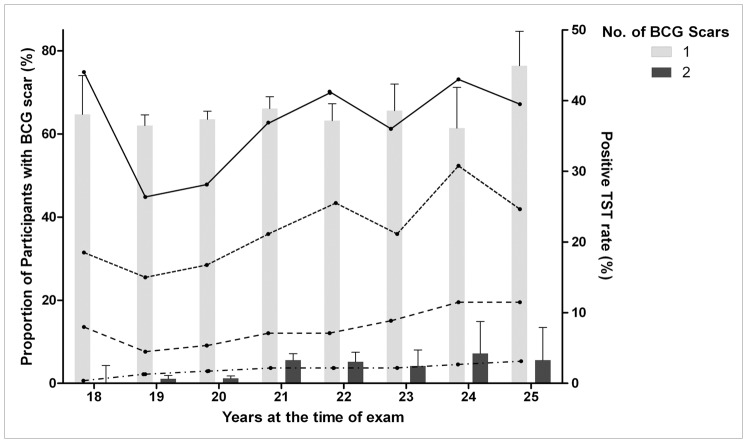
Number of BCG scars and TST-positive rates according to age at the time of examination.

TST positivity rates were higher for older participants. Exceptionally, the participants, with induration ≥5 mm, ≥10 mm and ≥15 mm, were more common in 18-year-olds than 19-year-olds. There was no significant difference in the number of BCG scars according to age (Figure 3).

## Discussion

The present study examined the TST results from 5,552 participants in Korea, a country with an intermediate TB burden that has experienced a recent and rapid change in BCG vaccination status. The results showed that TST positivity (defined as induration diameter of ≥10 mm) decreased from 28 to 15%. However, it is still not clear whether the rate of TB infection is really decreasing. This is because the trend of TST positivity may differ according to the definition of induration size for a “positive” response, and may be affected by the discontinuation of the BCG vaccination program in Korea.

Several factors may explain the epidemiologic change in the pattern of TST results. The TB incidence can affect the TST results because it can affect the frequency of its undetected TB exposure [13]. However, there was no significant change in the incidence of TB in Korea during the study period: 71.2/10^5^ in 2005, 68.9/10^5^ in 2008, 72.0/10^5^ in 2009, 71.9/10^5^ in 2010, and 78.0/10^5^ in 2011. We can confirm the TB incidence exactly after 2001, when the current TB notification system had settled, and the incidence between 2001 and 2004 was also similar (63.6–71.1/10^5^) [14], [15]. In this study, only 97 (1.7%) and 79 (1.4%) participants in the current study had a personal or family history of TB, respectively. Therefore, the TB incidence might not be the main factor of decrease in TST positivity rates.

BCG vaccination status is another important factor associated with a positive TST result [16], [17]. At present, there is no test that can reliably distinguish tuberculin reactions caused by BCG vaccinations from those caused by true TB infections. However, induration size and the number of years that have passed since the last vaccination can provide some information [18]. The association between a positive TST result and BCG vaccination has become less pronounced since larger induration sizes were used to define a positive test: indurations with a diameter ≥15–20 mm are unlikely to be caused by BCG [16], [19]–[21]. We found that the number of BCG scars was strongly associated with an induration diameter ≥5 or ≥10 mm, which is consistent with the results of previous studies [22]. Meanwhile, the number of BCG scars was not associated with a positive TST result defined by an induration diameter ≥15 or ≥20 mm. The time elapsed since the last BCG vaccination is another factor that can help to differentiate BCG responses from actual mycobacterial infections. The influence that the BCG vaccination has on the TST results declines gradually with time [19], reaching a nadir at 6–7 years post-vaccination (or 4 years in countries where the vaccine is given exclusively at birth) [23]–[25]. In the present study, participants of 18-years-old showed a higher positivity rate than participants of 19-year-old. This result may be due to BCG revaccination at 12 years-of-age because this increase in positivity for 18-year-olds was not observed if a positive TST result was defined as an induration diameter of ≥20 mm. BCG strain also can affect the TST results, but Pasteur was the only available strain in Korea, when the participants were vaccinated at their birth or 12-year-old [26].

Age is another factor that can affect the TST results [23]. However, the median age of the study participants remained steady throughout the study period and age was excluded from final multivariate analysis due to multi-colinearity with the year of birth, which was associated with BCG vaccination status.

Considering these factors which can affect TST results, it is difficult to suggest that the overall decrease in the rate of TST positivity in Korea is due to the successful control of LTBI. Although the overall TST positivity rate decreased, the percentage of participants with induration ≥20 mm in diameter did not decrease. The incidence of active TB in Korea remained steady, and there is no evidence to suggest that the incidence of NTM infection is decreasing [27]. As mentioned above, neither age nor a history of TB exposure had a major effect on positive TST rates. Therefore, the recent decrease in positive TST results for young Koreans might mainly be due to BCG vaccination status. It is interesting that a change in the national BCG vaccination strategy can have such an effect on TST results in a large population. This WHO recommendation (revaccination at 12-year-old be abandoned) could have been adopted in many countries, although the potential effects have not been evaluated. Without careful analysis of potential influential factors, the results of an epidemiologic survey based on TSTs can be misinterpreted, possibly leading to inappropriate TB control strategies.

The TST results for participants with no BCG scars or history of TB were similar to those of the other participants. One possible explanation for this is that these patients may have received a BCG vaccination that left no scar. The characteristic raised scar that results from a BCG vaccination is often used as a proof of prior immunization; however, the link between a post-vaccination scar and protection against the disease has yet to be determined [28]. About 90% of Koreans are given the BCG vaccination because it is both free and mandatory [29]. Despite this, 30% of those born after 1985 have no BCG scar. Less than 60% have a detectable scar at 3 years after vaccination; indeed, it is less likely that a BCG vaccination given in infancy will leave a lasting scar [30]. Participants born after 1985 may have received a single BCG vaccination during infancy due to the change in the vaccination strategy adopted by Korea. Meanwhile, those born before 1985 were revaccinated if the TST was negative; 90% of these individuals bare a BCG scar. This suggests that the absence of a BCG scar is not a clear indication that an individual has never been vaccinated with BCG.

This study has several limitations. First, the exact time of BCG vaccination could not be confirmed. BCG vaccination at an older age has a more persistent effect on TST results; thus, it is possible to estimate the effects of BCG more accurately if the exact time of vaccination is known. Instead, we can only speculate that participants with two BCG scars were vaccinated at birth and then again at 12 years-of-age. Participants with one or no BCG scars are likely to have been vaccinated at birth. A previous study suggests that BCG vaccination cards are not reliable, and that only the presence of a BCG scar should be used when analyzing the effects of BCG vaccines [23]. Second, the reporting of a personal or family history of TB is dependent upon the participants’ knowledge of/ability to remember the event. TB is treated with a 6 month course of medication; therefore, most participants should remember this. However, accounts of family history may not be accurate, leading to an underestimation. Third, it can be ambiguous if this study population is really representative of the general population. However, we can suggest that this population has almost same characteristics of general population of that age. This can be explained by several points. The military conscripts were recruited from all across Korea and a term of entire intake of military conscripts was enrolled without exclusion. Korea has mandatory military system; therefore all people without physical disability should join into army. Furthermore, we performed all examination including chest X-ray and TST on the next day of their joining into army. Therefore, the effect of communal setting is minimal.

In conclusion, the overall TST positivity rate in Korea has decreased (to 5–25%). However, this decrease seems associated with BCG vaccination status, rather than real decrease of LTBI prevalence. The present study addresses important issues regarding the interpretation of TST surveillance, which can lead TB control strategy in wrong way without careful interpretation. Changes in BCG vaccination strategy may raise similar issues in other countries; the data presented in the present study may be useful for future epidemiologic studies for TB control.
